# LncCeRBase: a database of experimentally validated human competing endogenous long non-coding RNAs

**DOI:** 10.1093/database/bay061

**Published:** 2018-06-21

**Authors:** Cong Pian, Guangle Zhang, Tengfei Tu, Xiangyu Ma, Fei Li

**Affiliations:** 1 Ministry of Agriculture Key Laboratory of Molecular Biology of Crop Pathogens and Insects, Institute of Insect Science, Zhejiang University, 866 Yuhangtang Road, Hangzhou, China; 2 Department of Mathematics, College of Science, Nanjing Agricultural University, Nanjing, Jiangsu, China; 3 Network Security Research Group, Institute of Network Technology, Beijing University of Posts and Telecommunications, Beijing, China; 4 School of Life Science, Anhui Agriculture University, Hefei, China

## Abstract

Long non-coding RNAs (lncRNAs) are endogenous molecules longer than 200 nucleotides, and lack coding potential. LncRNAs that interact with microRNAs (miRNAs) are known as a competing endogenous RNAs (ceRNAs) and have the ability to regulate the expression of target genes. The ceRNAs play an important role in the initiation and progression of various cancers. However, until now, there is no a database including a collection of experimentally verified, human ceRNAs. We developed the LncCeRBase database, which encompasses 432 lncRNA–miRNA–mRNA interactions, including 130 lncRNAs, 214 miRNAs and 245 genes from 300 publications. In addition, we compiled the signaling pathways associated with the included lncRNA–miRNA–mRNA interactions as a tool to explore their functions. LncCeRBase is useful for understanding the regulatory mechanisms of lncRNA.

## Introduction

The majority of sequences in the human transcriptome are classified as lncRNA (long non-coding RNA). When compared with genes encoding proteins and small molecule RNAs (such as miRNA), the number of lncRNAs is greatest (1–3), and their regulatory mechanisms are more diverse and extensive (4). Pre-existing evidence has shown that lncRNAs can regulate the expression of genes by interacting with proteins, RNA and DNA (5).

LncRNAs are directly involved in the regulation of gene expression and can affect an abundant number of target genes by interacting with sponging miRNAs (6). Although the structures of most lncRNAs and mRNAs are very similar, the regulation patterns of gene expression are more diverse and wide for lncRNAs. Increasing evidence has indicated that lncRNAs play critical roles in the biological processes of cancers (7, 8). Additionally, many studies have shown that competing lncRNAs play an important role in the initiation and progression of many cancers (9–11). LncRNAs have important potential applications, including prospects for new diagnostic methods and the treatment of malignant tumors (12).

Some lncRNA databases have been constructed, including lncRNAdb (13), lncRNAWiki (14), NONCODE (15) and LNCipedia (16). Additionally, there is the DIANA-LncBase (17), which integrates miRNA–lncRNA associations. Furthermore, the databases, LncRNADisease (18), lncRNASNP (19) and LincSNP (20) are a collection of relationships between lncRNAs and diseases. These databases are crucial for exploring the functions of lncRNAs in complex diseases in humans.

Only a limited number of lncRNAs have been validated by molecular experimentation. The experimentally verified lncRNAs are highly reliable and are important references for understanding the functions of lncRNAs. However, there is no database that is devoted to collecting experimentally verified competing endogenous RNAs (ceRNAs) (lncRNA–miRNA–mRNA). Here, we developed a database (LncCeRBase) to collect experimentally supported lncRNA–miRNA–mRNA interactions. All of the triplet interactions in the LncCeRBase were manually curated from published literature. The LncCeRBase database contains 432 lncRNA–miRNA–miRNA interactions, including 130 lncRNAs, 214 miRNAs and 245 genes from 300 publications. The LncCeRBase database should be helpful in understanding the regulatory mechanisms of lncRNA in complex diseases.

## Data sources and implementation

In constructing the database, scientific publications were obtained through searching keywords such as ‘lncRNA’, ‘ceRNA’, ‘competing RNA’, ‘lncRNAs targeting’ ‘lncRNA targeting’, ‘miRNA sponges’ and ‘circRNAs as miRNA sponges’ in the PubMed database of the National Center for Biotechnology Information (NCBI). Then, we selected the resulting literature describing lncRNA–miRNA-mRNA triplet interactions. All of the selected lncRNA–miRNA-mRNA interactions were experimentally confirmed by utilizing RNAi, Western blots, qRT-PCR or luciferase reporter assays.

In addition, many lncRNAs can activate or inhibit signaling pathways as ceRNAs by sponging miRNAs (21, 22). Therefore, we compiled the signaling pathways of the mRNAs involved in lncRNA-miRNA-mRNA associations. Finally, the LncCeRBase database contains 432 lncRNA-miRNA-miRNA interactions, including 130 lncRNAs, 214 miRNAs and 245 genes from 300 publications.

MongoDB is a free and open-source cross-platform document-oriented database program. When compared with the classic mySQL, MongoDB has the following five advantages: (i) Weak consistency; (ii) The way the document structure is stored, the data can be accessed more easily; (iii) The built-in GridFS supports larger capacity storage; (iv) Built-in Sharding; and (v)Third parties are rich in support (this is the advantage of MongoDB compared with other NoSQL) (23). Therefore, all data in the LncCeRBase were stored and managed using MongoDB (version 3.2.). The web interfaces were built-in Python (version 3.5). The data processing programs were written in Python (version 3.5), and the web services were built using Nginx.

## Web interface

The web service, LncCeRBase, is available at http://lnccerbase.it1004.com. Users can browse the lncRNA names, miRNA names, mRNA gene names or diseases. When selecting an lncRNA, miRNA or mRNA in the ‘Browse’ page of the web site, the LncCeRBase will return a list of matched lncRNA-miRNA-mRNA triplet associations, containing the name (lncRNA, miRNA, and mRNA), PubMed ID, associated disease/tissue, description, title and pathway name. For every entity, we link the name of lncRNA, miRNA and mRNA to the resource of RNAcentral (24), miRBase (25) and NCBI gene (26) respectively. Besides, all data from the LncCeRBase database can be downloaded. Since a gene may have other names, we design the search section; users can determine lncRNA-miRNA-mRNA triplet associations by inputting any name of a gene.

## The application of LncCeRBase

LncCeRBase provides a user-friendly interface to conveniently browse, search and download data. With the rapidly increasing interest in ceRNA, LncCeRBase will significantly improve our understanding of lncRNA–miRNA–mRNA triplet associations in diseases and has the potential to be a valuable resource.

## Future directions

The LncCeRBase database will be updated with new experimentally supported lncRNA-miRNA-miRNA interactions every two months. We found that 90.5% (391/432) of the included lncRNA–miRNA–miRNA interactions were verified between 2016 and 2017. This phenomenon shows that the regulatory mechanisms of competing endogenous lncRNAs have recently been gaining increasing attention. Undoubtedly, there will be many studies regarding competing endogenous lncRNAs in the future. In recent years, several ceRNA prediction methods were proposed. For example, Sardina *et al.* developed a computational method, called CERNIA, which takes into account insights from *in vivo* and *in silico* experiments, such as 5’ UTR and coding region binding sites, and tissue-specific gene expression profiles, to uncover novel ceRNAs, by taking into account both validated and high-confidence miRNA–target interactions (27). Zarringhalam *et al.* predicted the ceRNA network of PTEN by calculating a set of probabilistic features (28). Zhang *et al.* proposed a multi-step method called miRSCoPPI to infer miRNA sponge co-regulation of protein–protein interactions in the breast cancer (29). However, the studies of ceRNA prediction algorithm are limited. We are also developing an algorithm to predict lncRNA–miRNA–miRNA interactions by constructing an lncRNA–miRNA–mRNA network. With the generation of large-scale RNA-Seq data from TCGA (The Cancer Genome Atlas), increasing lncRNA–miRNA–miRNA interactions will be discovered. This tool will be based on mRNA, lncRNA and miRNA expression data found in TCGA and will be integrated into the LncCeRBase in the near future.

## Discussion and conclusion

Here, we developed a database (LncCeRBase) to collect experimentally supported lncRNA–miRNA–mRNA interactions. The LncCeRBase database integrates this triplet interaction data and can help us to explore the regulatory mechanisms of lncRNAs. With the increasing attention and deepening of research on lncRNA genes, an increasing number of new lncRNAs have been discovered. Currently, the functions of many lncRNAs are unknown, and the function of lncRNAs as ceRNAs is a research area that has been even less explored. Therefore, the lncRNAs, which have a biological function as ceRNAs, deserve investigation.

## Funding

This work was supported by the National Key Research and Development Program (2016YFC1200600, 2017YFC1200602).

*Conflict of interest*. None declared.

## Notes

Citation details: Pian,C., Zhang,G., Tu,T. *et al.* LncCeRBase: a database of experimentally validated human competing endogenous long non-coding RNAs. *Database* (2018) Vol. 2018: article ID bay061; doi:10.1093/database/bay061

## Notes

Database URL: http://www.insect-genome.com/LncCeRBase
