# LncCeRBase: a database of experimentally validated human competing endogenous long non-coding RNAs

**DOI:** 10.1093/database/baz090

**Published:** 2019-06-25

**Authors:** Cong Pian, Guangle Zhang, Tengfei Tu, Xiangyu Ma, Fei Li

**Affiliations:** 1 Ministry of Agriculture Key Laboratory of Molecular Biology of Crop Pathogens and Insects, Institute of Insect Science, Zhejiang University, 866 Yuhangtang Road, Hangzhou, China; 2 Department of Mathematics, College of Science, Nanjing Agricultural University, Nanjing, Jiangsu, China; 3 Network Security Research Group, Institute of Network Technology, Beijing University of Posts and Telecommunications, Beijing, China; 4 School of Life Science, Anhui Agriculture University, Hefei, China

This manuscript has been corrected to update the LncCeRBase web address to http://www.insect-genome.com/LncCeRBase

